# Eccrine Nevus in the Forearm of a 16-Year-Old Presenting as Unilateral Hyperhidrosis: A Clinicopathological Correlation Paradigm

**DOI:** 10.3390/dermatopathology8030047

**Published:** 2021-09-18

**Authors:** Alejandro Martin-Gorgojo, Ignacio Sanchez-Carpintero, Ricardo Ruiz-Rodriguez, Ana-Belen Enguita-Valls

**Affiliations:** 1 Dermatology Department, Clínica Dermatológica Internacional, 28001 Madrid, Spain; ignacio@clinicacdi.com (I.S.-C.); ricardo@clinicacdi.com (R.R.-R.); 2 Pathology Department, Clínica Dermatológica Internacional, 28001 Madrid, Spain; abenguita@hotmail.com

**Keywords:** eccrine glands, hyperhidrosis, botulinum toxins, type A, neoplasms, adnexal and skin appendage, pathology, clinical, dermatology

## Abstract

A case of a purely eccrine nevus in an adolescent patient presenting with focal hyperhidrosis on an area comprising the left forearm and the dorsal aspect of the left hand is described. No clinically evident lesions were identifiable. Dermatopathologic findings were subtle, showing only a slight increase in the number of eccrine glands. Clinicopathological correlation was paramount to achieve the diagnosis.

## 1. Introduction

Hyperhidrosis is a skin disorder characterized by excessive sweating. It can affect at general, regional, or focal levels. It may have a primary origin, with excessive diaphoresis generally appearing in localized and symmetrical areas, such as axillae, palms, soles, or craniofacial region. Alternatively, it might be secondary to multiple causes, having more frequently a generalized involvement. Some series have quantified primary cases at more than 90%, with secondary cases being infrequent. Regarding the age of onset, most patients initiate excessive sweating between 14 and 25 years of age. However, given the full functionality of the eccrine glands from birth, it can be observed in infants and children [1].

Uncommon causes of secondary focal hyperhidrosis include eccrine nevi. These are considered hamartomas, characterized by hyperplasia and/or hypertrophy of eccrine glands. Eccrine nevi usually affect the forearms (but may have other locations, such as the forehead, the trunk, upper or lower extremities) and present during childhood or adolescence [2]. The anatomical preference for the forearm has been proposed to be related phylogenetically to the antebrachial organ of the lemur catta [3].

The diagnosis of this entity is based on clinical suspicion followed by pathological confirmation. In this manuscript, we present a case highlighting the importance of this clinicopathological correlation.

## 2. Case Presentation

A 16-year-old female patient with no relevant past medical history came to our clinic for evaluation of excessive sweating on an area comprising the left forearm and the dorsal aspect of the left hand. This focal hyperhidrosis started when the patient was four years old, disturbing her during the colder seasons (from November to February, which may be explained by the fact that the patient more frequently wore long-sleeved clothing), and worsened with stressful situations.

**Figure 1:**
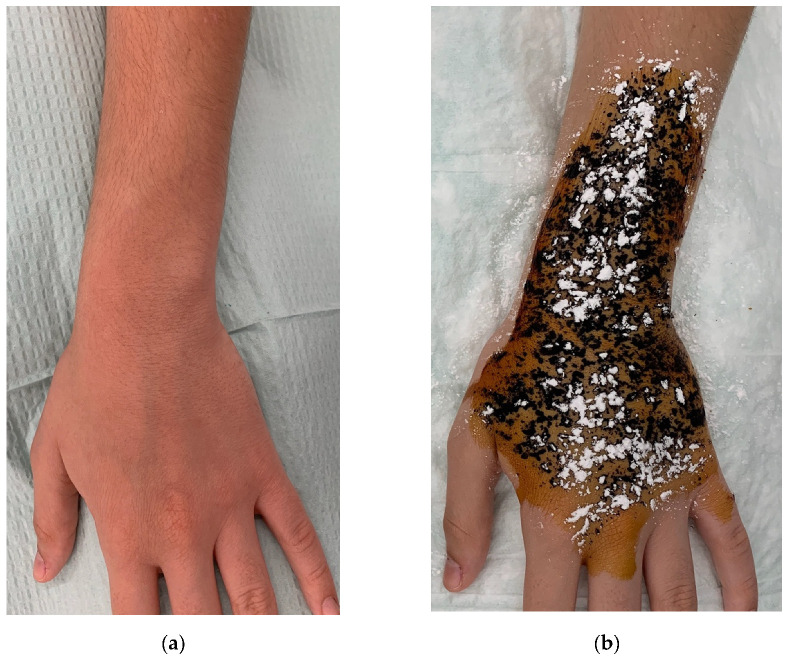
(**a**): Dorsal aspect of the patient’s left forearm, showing no clinically evident abnormalities. (**b**): Iodine-starch test (Minor’s test) highlighting the hyperhidrosis area.

Physical examination revealed no apparent cutaneous lesions (Figure 1a). The starch-iodine test highlighted the area of hyperhidrosis (Figure 1b).

**Figure 2:**
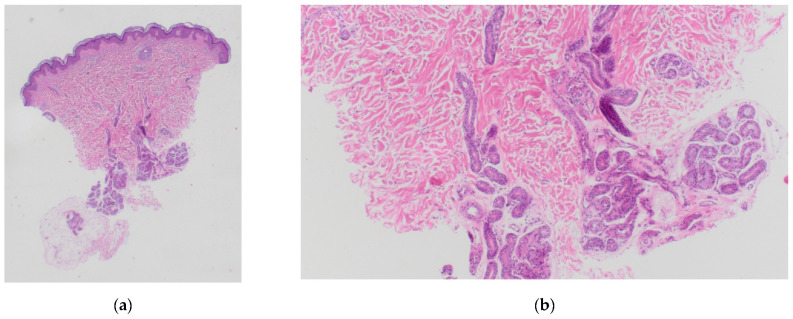
Punch biopsy sample, hematoxylin-eosin staining. (**a**): 10× magnification. (**b**): 40× magnification.

A punch biopsy was performed. The biopsy showed a slightly increased density of eccrine glands within the dermis, with no other abnormalities (Figure 2).

The patient was treated with multiple intradermal injections of botulinum toxin type A on the affected area, with a satisfactory response, remaining with anhidrosis seven months after follow-up.

## 3. Discussion

Eccrine nevi are rare lesions included in the group of organoid nevi, with few reported cases in the literature. Clinical features of eccrine nevi are variable, including their age of presentation, number, location, size, signs, and symptoms [4,5].

Most of the cases are only clinically evident when excessive sweating appears, as happened in our case. In some instances, it can be challenging to distinguish eccrine nevi from some forms of idiopathic localized unilateral hyperhidrosis. This hyperhidrosis is focal, on sharply demarcated areas of less than 10 × 10 cm in size, and occurs in short episodes frequently worsened by stress [4,6]. However, eccrine nevi may be identified as variably pigmented papules, depressed brown patches, solitary pores draining mucoid material, nodules [7], brownish scaly patches [8], or exceptionally as skin tags [9].

Histopathologically, purely eccrine nevi—such as the depicted case—are considered infrequent [10]. They present with subtle findings, comprising hyperplasia and/or hypertrophy of eccrine sweat glands. Other related organoid nevi may present with additional abnormalities apart from the increase in eccrine glands:

- Angiomatous eccrine nevi (or sudoriparous angiomas) show an augmented number of capillaries [4].
- Eccrine-pilar angiomatous hamartoma comprises hair follicles associated with the eccrine angiomatous complexes [7].
- Mucinous eccrine nevi have abundant mucin deposits around the increased number of ducts of the eccrine glands [11,12].

There are multiple options to treat eccrine nevi. Conservative management is an option if they remain asymptomatic or with mild symptoms. Topical antiperspirants such as aluminum salts plug the eccrine sweat pores, leading to the degeneration of secretory cells. Anticholinergic drugs such as glycopyrrolate work by competitively blocking the activation of eccrine glands. These may yield positive therapeutic results but usually need repeated applications [2] and are insufficient in some cases [13]. Therefore, botulinum toxin type A (which exerts its effect by inhibiting acetylcholine release and thus blocking the activation of eccrine glands) is a reasonable therapeutic option that can lead to satisfactory responses [5].

## 4. Conclusions

A case of a purely eccrine nevus in an adolescent patient presenting with focal hyperhidrosis has been described. Dermatopathologic findings were subtle, and clinicopathological correlation was paramount to achieve the diagnosis.
